# Initial Results after the Implementation of an Edge-To-Edge Transcatheter Tricuspid Valve Repair Program

**DOI:** 10.3390/jcm10184252

**Published:** 2021-09-19

**Authors:** Pedro Luis Cepas-Guillen, Juan Carlos de la Fuente Mancera, Joan Guzman Bofarull, Marta Farrero, Ander Regueiro, Salvatore Brugaletta, Cristina Ibañez, Laura Sanchis, Marta Sitges, Manel Sabate, Xavier Freixa

**Affiliations:** 1 Cardiology Department, Cardiovascular Institute (ICCV), Hospital Clinic, IDIBAPS, University of Barcelona, 08036 Barcelona, Spain; cepas@clinic.cat (P.L.C.-G.); delafuentemancera@gmail.com (J.C.d.l.F.M.); JGUZMAN@clinic.cat (J.G.B.); mfarrero@clinic.cat (M.F.); AREGUEIR@clinic.cat (A.R.); SABRUGAL@clinic.cat (S.B.); LSANCHIS@clinic.cat (L.S.); MSITGES@clinic.cat (M.S.); MASABATE@clinic.cat (M.S.); 2 Anesthesiology Department, Hospital Clinic, IDIBAPS, University of Barcelona, 08036 Barcelona, Spain; CRIBANEZ@clinic.cat

**Keywords:** tricuspid regurgitation, transcatheter valve repair, heart failure

## Abstract

Transcatheter tricuspid valve repair (TTVr) has emerged as an alternative for the treatment of severe tricuspid regurgitation (TR). We report our initial experience with an edge-to-edge TTVr system in a high-volume institution. Methods: We included consecutive patients who underwent edge-to-edge TTVr systems. The primary efficacy endpoint was a reduction in the TR of at least one grade. The primary safety endpoint was procedure-related clinical serious adverse events. Results: A total of 28 patients underwent TTVr with edge-to-edge systems. All patients presented with at least severe TR with a high impact on quality of life (82% of patients in NYHA class ≥ III). The Triclip system was the most used device (89%). The primary efficacy endpoint was met in all patients. Only one patient experienced a procedural complication (femoral pseudoaneurysm). At three-month follow-up, 83% of patients were in NYHA I or II (18% baseline vs. 83% 3 months follow-up; *p* < 0.001). Echocardiography follow-up showed residual TR ≤ 2 in 79% of patients (paired *p* < 0.001). At the maximum follow-up (median follow up = 372 days), no patients had died. Conclusions: Edge-to-edge TTVr systems seem to represent a very valid alternative to prevent morbidity and mortality associated with TR as depicted by the favorable efficacy and safety.

## 1. Introduction

Tricuspid regurgitation (TR) has been the subject of increasing interest in recent years, mainly because of its association with an increased risk of mortality and morbidity [1,2,3]. Currently, up to 86% of tricuspid valve surgeries are performed during left-sided heart disease interventions [1] with isolated tricuspid valve (TV) surgery being a rare procedure with an in-hospital mortality of nearly 10% [4]. Operative mortality risk related to right ventricular dysfunction and a high comorbidity burden are usually the main reasons why patients are turned down for surgery. Recently, transcatheter tricuspid valve repair (TTVr) techniques have emerged as an alternative for the treatment of severe TR in patients ineligible for surgery. These techniques seem to offer symptomatic improvement and a reduction in heart failure related hospitalizations with a low rate of complications [2,5,6]. The aim of the present study is to report the initial experience of edge-to-edge TTVr in our institution.

## 2. Materials and Methods

### 2.1. Patient Selection and Follow-Up

This observational study included all consecutive patients who underwent edge-to-edge TTVr in a high-volume institution between 2018 and 2021. The indication for TTVr was agreed upon by Heart Team at our institution, which reviewed the previous medical history and tricuspid valve anatomy to make the final decision regarding the indication of the procedure. All procedures were performed in a cardiac catheterization laboratory, and patients were under general anaesthesia using transoesophageal echocardiogram and fluoroscopic guidance. Preprocedural transthoracic and transoesophageal echocardiography were performed in all patients for assessing TR severity and suitability for edge-to-edge device implantation. Patients were treated predominantly with the Triclip system [7] but Mitraclip was also used in a compassionate and/or off-label use [8]. Details regarding the TTVr procedure and special features of the edge-to-edge device have been published elsewhere [9]. The follow-up protocol included a medical visit three months and one year after hospital discharge and a review of medical records at maximum follow-up. The first imaging follow-up with transesophageal echocardiography (TEE), was performed between the 10th and 14th week. All data were included in a prospective way involving a systematic review to look for inconsistencies or a lack of data. Data collected included demographic characteristics, medical history, baseline clinical characteristics, echocardiographic findings, procedural characteristics and in-hospital clinical and follow-up outcomes. The study was approved by the Institutional Committee and all subjects gave consent. The study conformed to the guiding principles of the Declaration of Helsinki.

### 2.2. Study Endpoints and Definitions

Standardized definitions of all patient-related variables, clinical diagnoses, and in-hospital complications and outcomes were used. Tricuspid regurgitation was assessed using standard 2D colour Doppler methods and graded using a five-class grading scheme: 0, none or trace; 1, mild; 2, moderate; 3, severe; 4, massive; and 5, torrential [10]. Implant success was defined as successful delivery and deployment of at least one clip with achievement of leaflet approximation and retrieval of the delivery catheter.

Following the landmark trials of the therapy, the primary efficacy endpoint was the reduction of the TR of at least one grade at discharge [6]. The primary safety endpoint was the occurrence of procedure-related clinical serious adverse events that included: death, cardiac tamponade, emergent surgery, vascular complications, major bleeding, stroke, and myocardial infarction. Secondary efficacy outcomes included all-cause mortality, cardiovascular mortality, heart failure rehospitalization, the New York Heart Association (NYHA) class, TR reduction, endocarditis, and non-elective cardiovascular surgery for tricuspid valve during the follow-up. Secondary safety endpoints included procedure-related technical complications as partial detachment, total detachment, leaflet perforation, and chordae rupture.

### 2.3. Statistical Analysis

Categorical variables are presented as frequencies (percentages), assessing the differences by Chi-square test (or Fisher test when necessary). Continuous variables are presented as mean ± standard deviation or as a median (interquartile range). The Kolmogorov–Smirnov test was applied to ensure normal distribution. Paired *t* tests were used to analyse changes in continuous variables between baseline and follow-up visits and Friedman tests were used for paired nominal data. Follow-up was considered to terminate at the date of the last follow-up. Analyses were performed using SPSS software (V 19.0, IBM, Armonk, NY, USA).

## 3. Results

### 3.1. Patient Population

A total of 28 patients underwent TTVr with edge-to-edge systems between January 2018 and May 2021. The median patient age was 76.5 years (range between 71 and 81), and women were predominant (89%). Baseline clinical characteristics are summarized in Table 1. Atrial fibrillation was present in almost all patients (93%). The expected operative risk was elevated (median EUROSCORE II = 3.76, IQR = 1.99–7.03). All patients presented TR ≥ 3 (severe in 39%, massive in 50% and torrential in 11%) with a high impact on quality of life according to the baseline NYHA class (82% of patients in NYHA class ≥ III) and hospitalizations for heart failure within the previous year (40% of patients). Echocardiographic characteristics at baseline are presented in Table 2. Tricuspid regurgitation was predominantly functional (94%). Systolic pulmonary artery pressure (PAP), as assessed by right heart catheterization, was 30.5 mmHg (range between 24.5 and 36.5 mmHg).

### 3.2. Procedural Results

**Figure 1:**
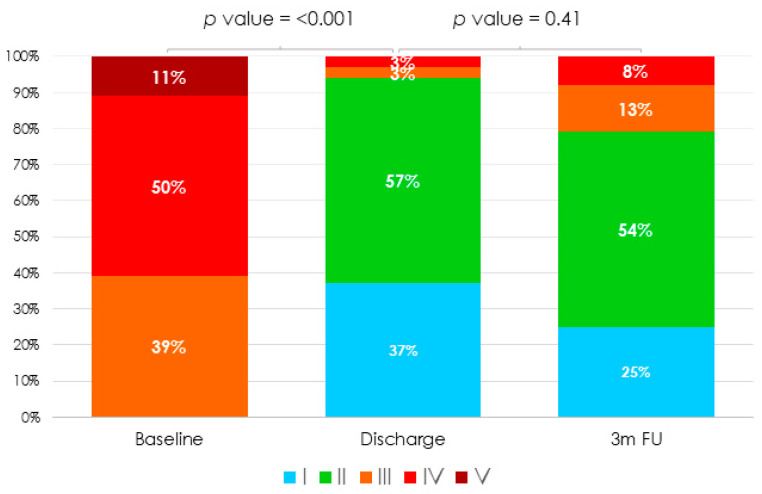
Tricuspid Regurgitation severity at baseline, discharge and three-month follow-up.

The implant was successful in 100% of patients. Procedural data are summarized in Table 3. The Triclip system was the most used device (89%). On average, one clip per patient was implanted (64%), with the use of two clips being less common (29%). The predominant clip position was anteroseptal (82%). Tricuspid valve mean gradient was 1.5 mmHg (0.8–1.6), with no cases of significant stenosis after therapy. Three patients presented a partial detachment. One detachment occurred during the intervention and could be fixed with the implantation of a second clip during the same procedure. The other two detachments were detected at follow-up. However, this complication was not related to any clinical adverse event. At discharge, all patients met the primary efficacy endpoint as defined by the presence of a TR reduction of at least one grade. In addition, the intervention decreased the proportion of patients with TR greater than severe from 100% pre-procedurally to 6% (*p* < 0.001) (Figure 1). Only one patient presented a procedure-related clinical adverse event: a vascular complication related to femoral access (pseudoaneurysm) that did not require any invasive treatment or surgery. The median length of stay after the procedure was two (1–3) days.

### 3.3. Clinical Outcomes at Follow-Up

**Figure 2:**
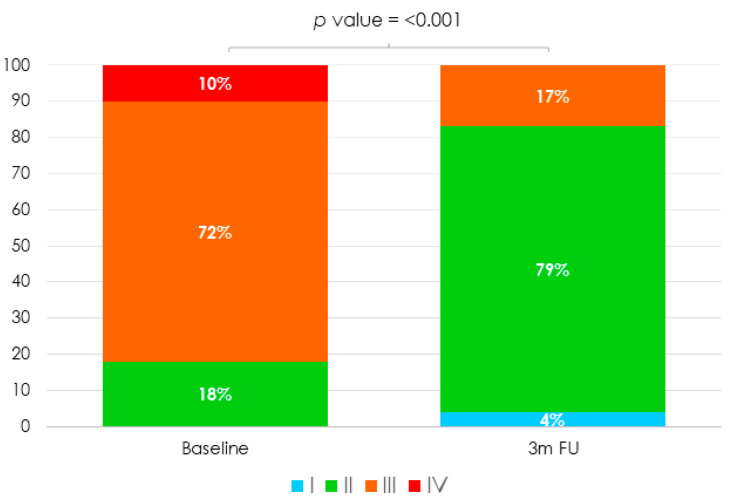
NYHA Functional Class at baseline and three-month follow-up.

Clinical outcomes at three months are provided in Table 4. Only one patient required hospitalization for HF. Most of patients (83%) were in NYHA I or II (Figure 2) (18% at baseline vs. 83% at three month follow-up; *p* < 0.001). Moreover, prescribed diuretic dose was reduced in 58% of patients. At three months, TTE showed residual TR ≤ 2 in 79% of patients (paired *p* < 0.001) (Figure 1). Although there was a slight increase in TR severity when comparing TTE at discharge and at three-month follow-up, no significant differences were observed.

At the maximum follow-up (median follow up = 372 days, IQR = 183–930), no patients had died. The total number of hospitalisations was also reduced compared to the pre-procedural period (13 baseline vs. 6 maximum follow-up; *p* = 0.023). One patient underwent tricuspid valve surgery (tricuspid valve replacement) due to the presence of persistent symptoms despite a reduction in the TR from torrential to severe after TTVr.

## 4. Discussion

The main findings of the present study, which assessed the initial experience with edge-to-edge TTVr systems, are: (1) patients undergoing edge-to-edge TTVr presented a high-risk profile and advanced stage of the disease; (2) edge-to-edge TTVr showed a high effectiveness in terms of TR reduction with a remarkable safety profile; (3) edge-to-edge TTVr was associated with excellent long-term outcomes, decreasing symptoms and hospitalization for HF.

The adverse effects of severe TR on long-term outcomes have been widely described, being associated with an increased risk of mortality and morbidity [3]. The natural history of TR leads to right-sided heart failure and recurrent hospitalization until refractory right heart failure and end-organ dysfunction appear [11]. Current treatment for TR is limited to optimal medical therapy, mainly involving diuretics, or surgery [12,13]. Of note, isolated TV surgery remains rare and continues to be associated with the highest surgical risk among all valve procedures in contemporary practice, with unchangeable operative mortality rates [14]. Despite the optimal medical treatment, patients with severe TR remain very symptomatic. This is something that can be seen in our cohort, where 82% of patients remained in NYHA class III or IV with congestive heart failure, oedema or/and ascites, despite intensive diuretic treatment. The high-risk nature of our patients is clearly seen by the fact that 40% had at least one HF hospitalization during the previous year. HF hospitalizations are not only associated with increased healthcare costs, but also with increased mortality [15]. The high number of HF hospitalizations might be explained not only by the increasing age and number of comorbidities, but also because of the progressive and self-perpetuating nature of TR and right heart dysfunction associated with limited therapeutic options beyond diuretics [16]. Regarding comorbidities, atrial fibrillation (AF) was extremely prevalent (93%) in our cohort. The prevalence of atrial functional TR is increasing and is associated with advanced age and right atrial enlargement. On the other hand, TV deformations and their association with right heart remodeling differ between atrial functional TR and left-sided heart disease-TR [17]. Atrial fibrillation patients with isolated TR showed more right auricular dilation and right ventricular conical remodeling than patients with AF and no TR. Furthermore, isolated TR was independently associated with worse long-term prognosis in terms of mortality, hospitalization for heart failure, and stroke compared with patients without TR [18].

In recent years, a growing body of knowledge around transcatheter valve technologies has emerged, widening the spectrum of patients who are referred to valve intervention [19]. Thus, TTVr has become an alternative to tricuspid surgery in patients with TR that were turned down for surgery. Our report describes the outcomes of 28 patients undergoing TTVr for symptomatic TR ≥ 3. Despite their high-risk profile and advanced stage of the disease (82% in NYHA class ≥ III), TTVr emerged as an effective and safe procedure in this population. Tricuspid regurgitation was reduced in all cases and the incidence of complications was very low. Our results are in agreement with previous studies: In the Transcatheter Tricuspid Valve Therapies (TriValve) registry [20], Mehr et al. evaluated procedural, and 1-year clinical and echocardiographic outcomes of patients treated with edge-to-edge TTVr. In 249 patients (mean age 77 ± 9 years), TR reduction to grade ≤ 2 was achieved in 77% by placement of 2 ± 1 clips, predominantly in the anteroseptal position. Predictors of procedural failure included effective regurgitant orifice area, tricuspid coaptation gap, tricuspid tenting area, and absence of central or anteroseptal TR jet location. Of note, this study only included patients treated with edge-to-edge TTVr in compassionate and/or off-label use as MitraClip NT and MitraClip XTR, whereas the use of MitraClip system in our study was residual (10%). More recently, the Transcatheter Edge-to-Edge Repair for Treatment of Tricuspid Regurgitation (TRILUMINATE) trial assessed the efficacy and safety of the TriClip transcatheter tricuspid valve repair system, a specific tricuspid edge-to-edge system and the most used device in our study [5,6]. Tricuspid regurgitation severity was reduced by at least one grade at 30 days in 86% of patients. Regarding safety data and similarly to our study, 7% presented single leaflet device detachment (three anterior, two septal) without relevant clinical impact. On the other hand, in six patients, mean gradient across the tricuspid valve was greater than 5 mm Hg, requiring no further intervention. No cases of stroke or device embolization were reported within six months.

In addition to the efficacy in terms of TR reduction and low rate of procedural MAEs, follow-up data showed the clinical benefit of the procedure after discharge, reducing the symptoms and the number of hospitalizations for HF. In our cohort, 83% of patients were in NYHA I or II at follow-up compared to 18% of patients at baseline. This improvement in NYHA functional class is correlated with a reduction in hospitalization ifor HF. While the total number of hospitalizations for HF was 13 in the year before the intervention, it was reduced to six in a similar period after the procedure, representing a 54% reduction in HF hospitalisations. In the TriValve registry, at one-year follow-up, significant and durable improvements in TR severity (TR ≤ 2 in 72% of patients) and NYHA functional class (≤2 in 69% of patients) were observed [20,21]. Better results were obtained when a TriClip system was used in the TRILUMINATE trial [5,6] as patients experienced significant clinical improvements in NYHA functional class I/II (31% to 83%, *p* < 0.0001), 6 min walk test (272.3 ± 15.6 to 303.2 ± 15.6 meters, *p* = 0.0023) and Kansas City Cardiomyopathy Questionnaire (KCCQ) score (improvement of 20 ± 2.61 points, *p* < 0.0001). Edge-to-edge TTVr also demonstrated a significant reverse right ventricular remodelling in terms of size and function. This observation is relevant since the impact of TTVr is not only exclusive to the TR but also to the right ventricular function. This could be explained by a reduction in chronic right ventricular volume overload without an increase in right ventricular afterload, which results in improved right ventricular performance, left ventricular filling, and cardiac output [22]. In addition, it would also explain the stability or even the improvement in the procedural results in terms of TR reduction at follow-up [7]. Although preliminary data about the use of TTVr in this challenging setting are favourable [21], compared to medical treatment, this approach needs to be validated in randomized clinical trials. The ongoing TRILUMINATE Pivotal trial (ClinicalTrials.gov Identifier: NCT03904147) should confirm this hypothesis.

The main limitation of this study is its observational design, which implies an inherent selection bias. Moreover, it is difficult to capture and control all potential confounders when using a registry. Therefore, our results should be considered hypothesis-generating. In addition, the sample size may lack power to detect predictors of improvement, such as tricuspid annulus reduction, TR reduction or right ventricular function, in NYHA class function. Larger randomized clinical trials are needed to fully clarify the best approach and characterize the actual potential benefit of TTVr in this high-risk cohort of patients.

## 5. Conclusions

Tricuspid regurgitation management remains a challenge due to high-risk patient profiles and limited therapeutic strategies. Edge-to-edge TTVr systems seem to represent a very valid alternative to prevent morbidity and mortality associated with TR as depicted by the favourable effective and safety outcomes. Larger registries and randomized trials are warranted to confirm this promising results.

## Figures and Tables

**Table 1:** Baseline clinical characteristics (N = 28).

Age, years	76.5 (71–81)
Female	25 (89)
Body mass index, kg/m^2^	26.7 ± 4.89
Hypertension	17 (61)
Diabetes mellitus	5 (18)
Atrial fibrillation	26 (93)
Chronic obstructive pulmonary disease	7 (25)
Coronary artery disease	2 (7)
Stroke/TIA	4 (14)
Estimated glomerular filtration rate	59.5 ± 23.6
PPM/ICD lead	1 (3)
Previous valve surgery - Mitral valve - Tricuspid valve	3 (10) 1 (4)
Previous transcatheter mitral valve intervention	2 (6)
EuroSCORE II, %	3.76 (1.99–7.03)
NYHA functional class - I - II - III - IV	0 20 (18) 22 (72) 3 (10)
Peripheral oedema	26 (93)
Ascites	12 (43)
Previous hospitalization for heart failure *	11 (40)
Number of admissions for heart failure *	13
Medical treatment - Betablockers - ACEI/ARA-II - Aldosterone Antagonists - Sacubitril/Valsartan - AVK - NOAC - Nitrates + Hydralazine - Furosemide - Thiazide	18 (64) 9 (32) 9 (32) 1 (4) 15 (54) 8 (29) 1 (4) 25 (90) 10 (36)

Values are n (%), mean ± SD or median (interquartile range). TIA = transient ischemic attack; PPM = permanent pacemaker; ICD = implantable cardioverter defibrillator; EuroSCORE = European System for Cardiac Operative Risk Evaluation; NYHA = New York Heart Association; ACEI = angiotensin converting enzyme inhibitors; ARAII = angiotensin II receptor antagonists. * In the previous 12 months.

**Table 2:** Echocardiographic and right heart catheterization characteristics.

Left ventricular ejection fraction, %	59 (55–60)
Left ventricular end-diastolic diameter, mm	44 (40–49.5)
Left ventricular end-systolic diameter, mm	27.5 (25–31)
Mitral regurgitation severity - No (0) - Mild (1) - Moderate (2) - Moderate-severe (3) - Severe (4)	3 (11) 21 (75) 3 (11) 1 (4) 0
Tricuspid regurgitation severity - None - Mild (1) - Moderate (2) - Severe (3) - Massive (4) - Torrential (5)	0 0 0 11 (39) 14 (50) 3 (11)
Tricuspid regurgitation aetiology - Functional - Degenerative - Mixed - Annulus size, mm	26 (94) 1 (3) 1 (3) 46.2±0.8
Pacemaker lead-associated tricuspid regurgitation	1 (3)
Echocardiography systolic PAP, mmHg	40 (35–47)
RHC systolic PAP, mmHg	30.5 (24.5–36.5)
RHC right ventricular peak pressure, mmHg	19 (14.5–21.5)

Values are n (%), mean ± SD or median (interquartile range). PAP = pulmonary artery pressure; RHC = right heart catheterization.

**Table 3:** Procedure data.

Implant success rate	28 (100)
Number of clips per patient - 1 - 2 - 3	18 (64) 8 (29) 2 (7)
Type of device - MitraClip - TriClip	3 (11) 25 (89)
Type of clip NT/XT (%) - NT - XT	15 (37) 25 (63)
First clip location - Anteroseptal - Posteroseptal - Anteroposterior	23 (82) 5 (18) 0
Second clip location - Anteroseptal - Posteroseptal - Anteroposterior	7 (64) 4 (36) 0
Post-procedure TV mean gradient, mmHg	1.5 (0.8–1.6)
Procedural time, min	130 (100–185)
Fluoroscopy time, min	26 (22–37)
Final in-lab TR severity - Mild (1) - Moderate (2) - Severe (3) - Massive (4) - Torrential (5)	16 (57) 12 (43) 0 0 0
Technical complications - No - Partial detachment - Total detachment - Leaflet perforation - Chordae rupture	25 (90) 3 (11) 0 0 0
Procedure-related clinical adverse events - No - Death - Cardiac tamponade - Emergent surgery - Vascular complications - Major bleeding - Stroke - Myocardial infarction - Permanent pacemaker implant - Intensive care unit stay - Inotropes treatment after TTVR - Others	27 (96) 0 0 0 1 (4) 0 0 0 0 0 0 0
Post-procedural length of hospital stay, days	2 (2–3)
Successful implantation	28 (100)
Pre-discharge TR severity - Mild - Moderate - Severe - Massive - Torrential	10 (37) 16 (57) 1 (3) 1 (3) 0
Antithrombotic strategy at discharge - None - Single antiplatelet therapy - Dual antiplatelet therapy - AVK - NOAC	1 (3) 1 (3) 2 (6) 12 (44) 12 (44)

Values are n (%), mean ± SD or median (interquartile range). TV = tricuspid valve; TR = tricuspid regurgitation; AVK = antivitamin K antagonist; NOAC = non-vitamin K antagonist oral anticoagulant.

**Table 4:** Outcomes.

**Clinical and Echocardiography Follow-Up at Three months**	
All-cause mortality	0
Cardiovascular mortality	0
Hospitalization for heart failure	1 (3)
NYHA functional class - I - II - III - IV	1 (4) 19 (79) 4 (17) 0
Decrease of ≥1 NYHA functional class	20 (77)
Diuretic dose modification - No - Yes, reduce - Yes, increase	9 (38) 14 (58) 1 (4)
Tricuspid regurgitation severity - Mild - Moderate - Severe - Massive - Torrential	6 (25) 13 (54) 3 (13) 2 (8) 0
**Clinical Outcomes at Last Follow-Up**	
Follow-up time, days	372 (183–930)
All-cause mortality	0
Cardiovascular mortality	0
Hospitalization for heart failure	4 (16)
Number of admissions for heart failure	6
Endocarditis	0
Tricuspid valve surgery	1 (3)

Values are n (%), mean ± SD or median (interquartile range). NYHA = New York Heart Association.
